# Biological activity and microscopic characterization of *Lythrum salicaria* L

**DOI:** 10.1186/2008-2231-21-61

**Published:** 2013-07-25

**Authors:** Azadeh Manayi, Mahnaz Khanavi, Soodabeh Saiednia, Ebrahim Azizi, Mohammad Reza Mahmoodpour, Fatemeh Vafi, Maryam Malmir, Farideh Siavashi, Abbas Hadjiakhoondi

**Affiliations:** 1 Department of Pharmacognosy, Faculty of Pharmacy, Tehran University of Medical Sciences, Tehran, Iran; 2 Traditional Iranian Medicine and Pharmacy Research Center, Tehran University of Medical Sciences, Tehran, Iran; 3 Medicinal Plants Research Center, Faculty of Pharmacy, Tehran University of Medical Sciences, Tehran, Iran; 4 Department of Pharmacology and Toxicology, Faculty of Pharmacy, Tehran University of Medical Sciences, Tehran, Iran; 5 Med.UL, Faculty of Pharmacy, University of Lisbon, Lisbon, Portugal; 6 Microbiology Department, Faculty of Sciences, University of Tehran, Tehran, Iran

**Keywords:** *Lythrum salicaria*, Antioxidant, Diabetes, *H. pylori*, Microscopy characterization

## Abstract

**Background:**

There are several plants have been used worldwide in the folk medicine with high incidence for treatment of human disorders, of which *Lythrum salicaria* belongs to the Lythraceae family has traditionally reputation for some medicinal usage and recently many biological and pharmacological activity of the plant have been studied.

**Methods:**

In this study, microscopic characterizations of the aerial parts of the plant were determined. Moreover, the plant extract (aqueous methanol 80%) was subjected to an anti-diabetic activity test (in a rat model of streptozocin induced diabetes), anti-*Helicobacter pylori* (using disc diffusion method) along with antioxidant activity against DPPH (stable free radical) tests. Besides, total flavonoids, phenols, tannins, as well as polysaccharides contents have been assessed using spectroscopic methods.

**Results:**

The microscopic properties of the plant fragments revealed anomocytic stomata, conical shape trichomes, and abundant spherical pollen grains as a characteristic pattern for the aerial parts of the plant. The extract of the plant at concentration of 15 g/kg showed mild lowering activity on blood glucose level to 12.6% and 7.3% after 2 and 3 h of administration. Additionally, clinically isolated *H. pylori* strain was inhibited with the plant extract at concentration of 500 mg/mL (zone of inhibition: 17 ± 0.08 mm). Moreover, IC_50_ values for DPPH inhibition of the plant extract, vitamin E, BHA were examined as 13.5, 14.2, and 7.8 μg/mL, respectively. Total flavonoids, phenols, tannin, and polysaccharides contents of the extract were successfully evaluated as 5.8 ± 0.4 μg QE/mg EXT, 331 ± 3.7 μg GAE/mg EXT, 340 ± 2.3 μg TAE/mg EXT, 21 ± 0.2 μg GE/mg EXT, respectively.

**Conclusions:**

The results suggested that *L. salicaria* has low anti-diabetic and anti-*Helicobacter pylori* effects, but high antioxidant activity, just the same as positive standard (vitamin E), which might be attributed to the high content of phenolic compounds in the extract.

## Background

Plants have performed a substantial role in traditional treatment or prevention of the complexity of diseases. They reduce the risk of many chronic diseases including diabetes, cancer, cardiovascular disorders and other sicknesses [1]. Identification of the plants powders is a complex criterion for herbalist. Although recognition of plant materials is one of the main concerns in phytotherapy, microscopical examination has been accepted as a standard technique for determination of herbal drugs in a powdered mixture [2].

*Lythrum salicaria* is a perennial herbaceous plant belonging to the Lythraceae family, which consists of 30 species. Seven of the mentioned species are growing wildly in Iran. This plant distributed in the north and north-west of Iran in the wetlands near streams [3,4]. The plant is well- known as “Turbinkwash”, “Yerpoose” and “Surmankhal” in Persian language [4]. Despite of the fact that this plant was considered as a wild invasive herb, its flowering aerial part has been used as a medicinal plant from ancient time. Aerial parts of the plant have been traditionally employed for treatment of diarrhoea, dysentery, inflammation of intestine, haematuria, leucorrhoea, epitaxis and dysmenorrhoea. Additionally, the plant used externally for cleaning impetigo, eczema, lupus and inflammation of female genito-urinary system in the north-west of Iran [5]. Scientific investigations of *L. salicaria* recently demonstrated antibacterial, antifungal, antioxidant, anti-inflammatory, anti-nociceptive, and cytotoxicity effects [6-10]. The literatures revealed that *L. salicaria* extracts showed decreasing activity on blood glucose level in both normoglycemic and glucose induced hyperglycemic rats as well as rabbits through augmentation of insulin secretion, and also it could reduce the plasma level of triglycerides [11,12]. Betulinic acid and its derivatives, isolated from ethyl acetate extract of the plant, exhibited suppressive activity on human Acyl-CoA: cholesterol acyl transferase (hACAT), suggesting that the plant might be useful for the hypercholesterolemia and atherosclerosis treatment and prevention [13]. Lately, anti-tussive and bronchodilator activity of the plant was determined due to polysaccharide-polyphenolic conjugate in the guinea pig [14]. Moreover, the high mass molecules (polysaccharide-polyphenolic conjugate) showed controversial anticoagulant and procoagulant effects in the preceding experiments [15,16]. Phytochemical purification of the plant have been revealed the presence of various bioactive compounds such as tannins, flavonoids, phenolic acids, anthocyanins, alkaloids, sterols, and triterpens in preceding studies [6,17-19]. It is also explored that there are three forms of plant flowers varying in both size and type of stigma, which facilitate legitimate pollination [20].

In the present study, microscopic characterization of the aerial parts of the plant as well as anti-diabetic, anti-*Helicobacter pylori*, and antioxidant effects of the aqueous methanol extract (80%) of *L. salicaria* have been assessed along with phytochemical contents of the extract. This is the first report of the microscopic structural characterization and anti-*Helicobacter pylori* activity of the plant followed by polysaccharides content.

## Methods

### Plant material

Aerial parts of *L. salicaria* were collected in May 2011 from Lahidjan city, Guilan province in the north of Iran and has been deposited at the Herbarium of Institute of Medicinal Plants, Jahade-Daneshgahi (ACECR), Karaj, Iran (Ajani 313). The cleaned plant materials were dried in the shade at room temperature and extracted with aqueous methanol (80%) using percolation apparatus.

### Powdered plant characterization

One gram of each tissue powder (flower, leave, and stem) of *L. salicaria* was heated in potassium hydroxide solution (10%) in a backer on heater for 30 seconds (flower and leave) or 1 minute (stem) depending on the tissue hardness, and washed afterwards with distilled water three times. The treated powders were sequentially washed with sodium hypochlorite for whitening and then rinsing with distilled water. The preparation was mounted in aqueous glycerin [2]. Photomicrographs were taken using Zeiss microscope attached with a digital camera. Photomicrographs of sections were captured at various magnifications conditional upon the microscopic details to be observed.

### Anti-diabetic test

### Animals

Male Wistar rats weighing about 190–200 g body weight were housed at standard laboratory conditions and fed with rodent pellet diet and water *ad libitum*. The animals were kept at room temperature and a photoperiod of 12 h day/night cycle. They considered as fasted were deprived of food for 14 h but had free access to water. The rats were obtained from animal house of Faculty of Pharmacy, Tehran University of Medical Sciences. All ethical manners for use of animals in scientific research have been carefully considered.

### Induction of diabetes

Streptozocin (STZ) was freshly prepared in normal saline (22 mg/mL). Type 2 diabetes mellitus (T2DM) was induced in overnight fasted rats by a single intraperitoneal injection of STZ (55 mg/kg). Hyperglycemia was confirmed polydipsia, polyuria and by the elevated glucose degree in plasma, calculated at 60 h after injection by glucometer. Rats with a blood glucose level above 250 mg/dL were choosing for the experiments.

### *In vivo* determination of anti-diabetic activity (T2DM model)

The animals (n = 24) were divided into four groups of six animals. The extract of the plant dissolved in DMSO that orally administered to the experimental groups (15 and 12.5 g/kg body weight) and a control group were also fed with DMSO (10 mL/kg). Previous investigation demonstrated that LD_50_ of DMSO (lethal dose, 50%) in rats is 20 mL/kg [21]. Glibenclamide (3 mg/kg) was administered to the diabetic rats as a positive control. Samples of blood were achieved from the tail at 1, 2 and 3 h after vehicle, samples and drug administration. Concentration of blood glucose was estimated by enzymatic glucose oxidase method using a commercial glucometer (Accucheck, Germany). The percentage variation of glycemia for each group was calculated in relation to initial (0 h) level, according to:

$$\%\mathrm{Variation}\;\mathrm{of}\;\mathrm{glycemia}=\left[\left(\mathrm{Gi}‒G0\right)/G0\right]\times 100$$

G0: initial glycemia values and Gi: glycemia values at 1, 2 and 3 h, respectively.

### Assay of anti-*Helicobacter pylori* effect

*Helicobacter pylori* were isolated from patients with chronic gastritis who had been introduced to the Endoscopy Unit at Shariati Hospital, Tehran, Iran. The biopsies from patient with antrum gastritis or peptic ulcer and positive rapid urea breath test were cultured on selective brucella agar under microaerobic situation. Isolated *H. pylori* were characterized using Gram stains, exhibited Gram negative spiral forms, positive urease, oxidase and catalase tests. The surface of brucella agar plus 7-8% blood plates were inoculated with 100 μL of bacterial suspension (bacterial suspensions in normal saline with the turbidity of McFarland standard No. 2 equivalent to 6 × 10^8^ cell/mL) and dried at room temperature for 10 min. Afterwards, the sterile blank disc (diameter: 6 mm) were impregnated in the plant extract (500 mg/mL) and placed on the plates, these plates were incubated at 37°C under microaerobic conditions and examined after 3–5 days [22]. Positive control plates included discs impregnated with amoxicillin (1 μg/mL) and the inhibition zone diameters (IZD) were recorded.

### Free radical scavenging activity assay

Free stable radical, 2,2-Diphenyl-1-picryl-hydrazyl (DPPH), has been widely used to evaluate the free radical scavenging activity of natural products. The plant extract activity against free radical was examined by DPPH method which described previously [23]. The absorptions at 517 nm were measured after 30 min. Free radical 50% inhibition (IC_50_) provided by extracts concentrations were calculated according to the plot of inhibition percentage against extracts concentration. Besides, vitamin E and BHA were used as positive standards.

### Assay of total flavonoids

The flavonoids content were measured at 425 nm by creating a complex with AlCl_3_ in a methanol-ethyl acetate-acetic acid medium [24]. The contents of total flavonoids, expressed as quercetin equivalent in the extract (μg QE/mg EXT), were calculated from following expression: (A × 0.875)/b

A: the absorbance of the test solution at 425 nm

b: the mass of the powdered extract in grams

### Assay of total phenols

Total phenolics content of the plant extract was examined and expressed as gallic acid equivalent (μg GAE/ mg EXT) using Folin Ciocalteu method [23]. The sample absorbance was compared to gallic acid calibration curve.

### Assay of total tannins

The method of total tannins could be coupled with the use of insoluble matrix, polyvinylpolypyrrolidone (PVPP) which binds with tannin-phenolics for measurement of tannins. The percentage of total tannins was calculated as tannic acid equivalent in dry extract (μg TAE/mg EXT) using tannic acid calibration curve (Y = 0.042X + 0.077, r^2^ = 0.974) [25].

### Determination of total polysaccharides

Total sugar (including polysaccharides) content or polysaccharides content was determined according to previous study [26]. In this method, all the glycosidic linkages in the presence of sulfuric acid are broken and a colored aromatic complex achieved between phenol and the carbohydrate, afterwards absorbance of this complex was measured. The samples absorptions were compared to calibration curve of different concentration of glucose (5–50 μg/mL) and the results reported as glucose equivalent in the extract (μg GE/mg EXT) using glucose calibration curve (Y = 0.014X + 0.024, R^2^ = 0.998).

### General

Substances (all by analytical grades) including solvents, glucose, gallic acid, Folin Ciocalteu, DMSO and brucella agar were purchased from Merck (Germany), along with Glibenclamide and STZ purchased from Tehran Chemistry (Iran) and Pharmacia and Upjohn (USA), respectively.

### Statistical analysis

All values were expressed as mean ± SE. Statistical significance was estimated by analysis of variance (ANOVA) following by Tukey post hoc test for multiple comparisons, p < 0.05 implies significance.

## Results and discussion

### Microscopic characterization

**Figure 1:**
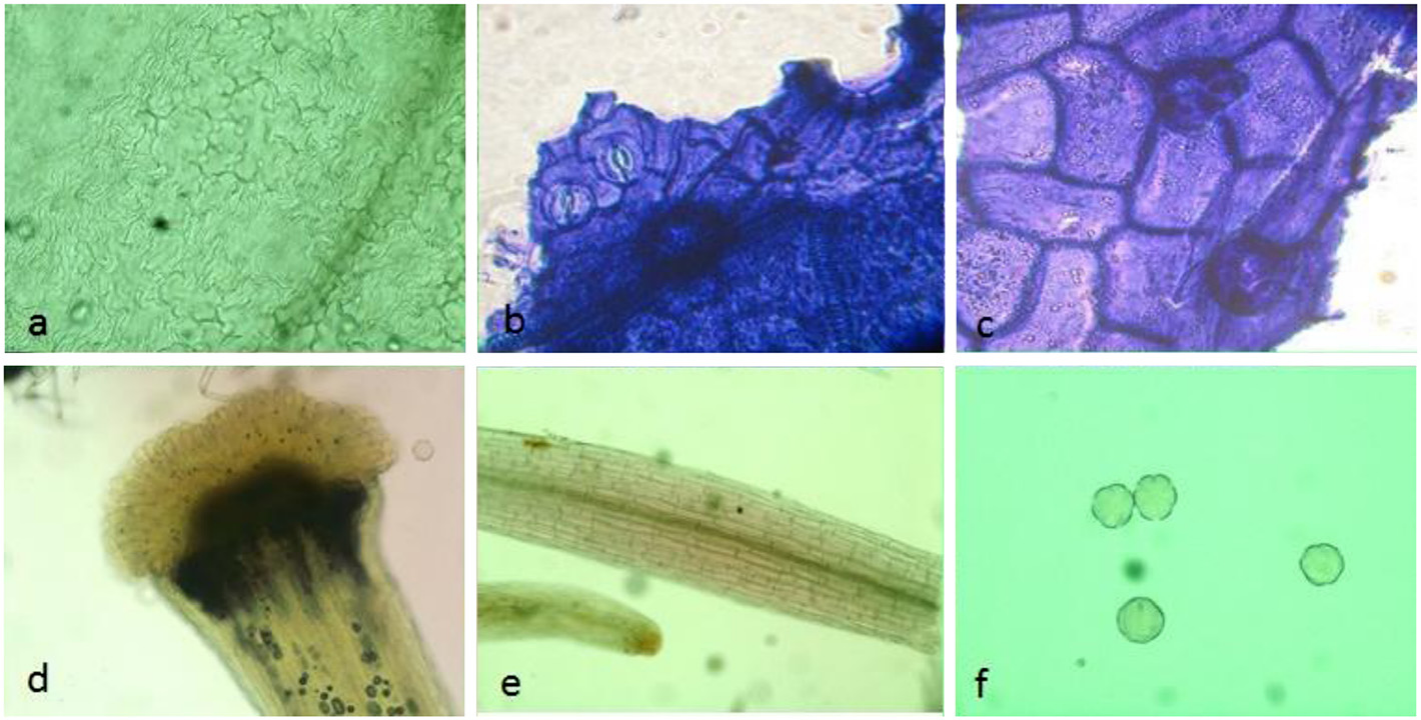
**Microscopic characterization of *L. salicaria *flower, a: corolla epidermis cells, b: anomocytic stomata of calyx, c: cicatrix of calyx, d: papillae of stigma and style, e: anther in surface view, f: pollen grains.**

**Figure 2:**
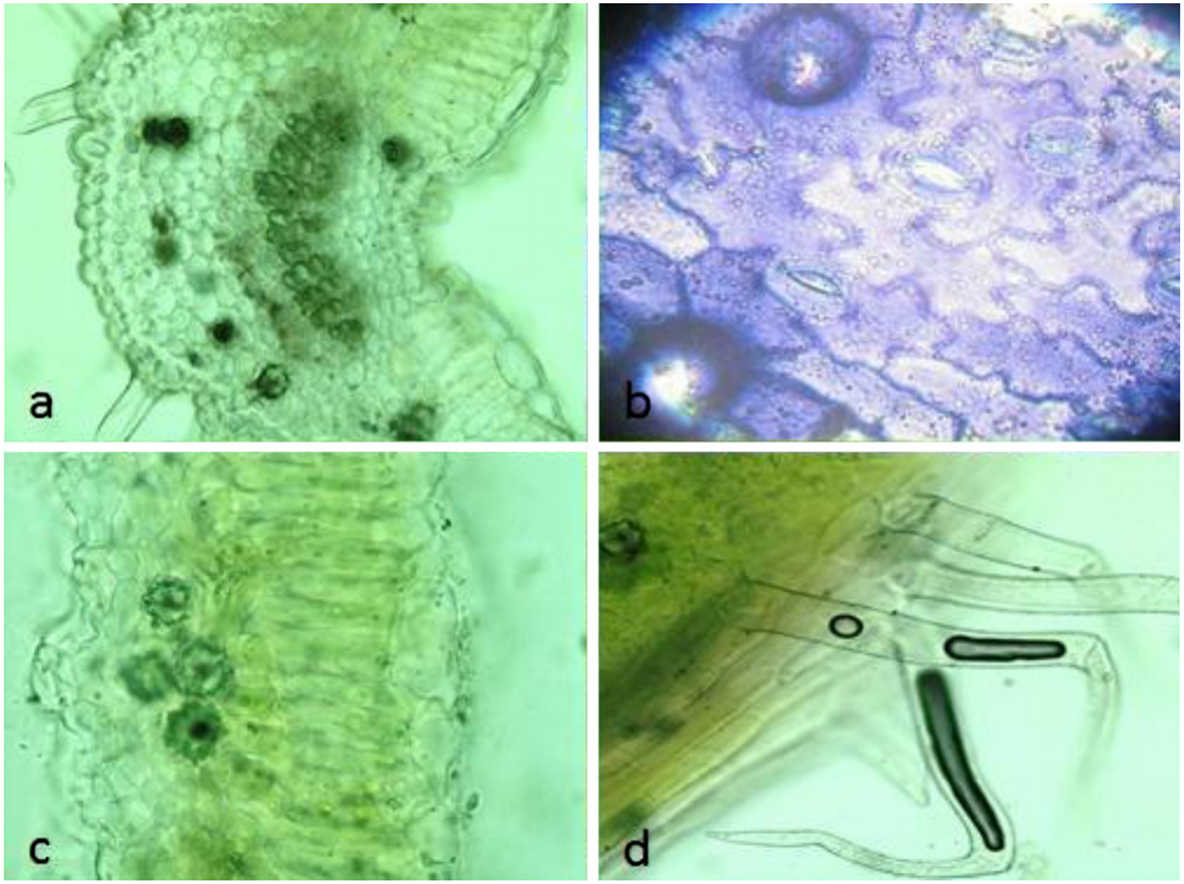
**Microscopic characterization of *L. salicaria *leaf, a: leaf in the sectional view, b: epidermis with sinuous cell walls, anomocytic stomata and cicatrix, c: upper and lower epidermises, palisade cells and mesophylls containing calcium oxalate clusters, d: covering trichomes.**

**Figure 3:**
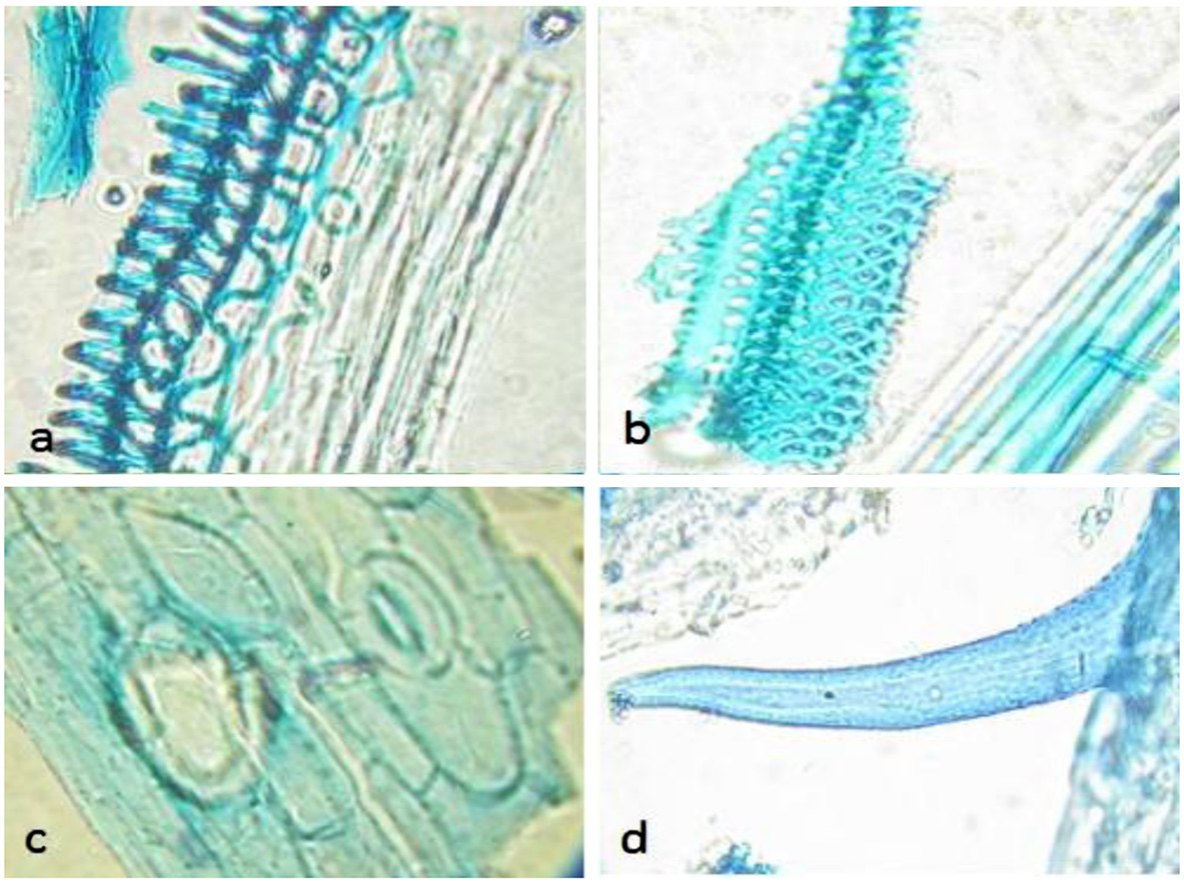
**Microscopic characterization of *L. salicaria *stem, a: vessels of the stem with spiral thickening, b: vessels with reticulate thickening and fibrous layer, c: anomocytic stomata and cicatrix, d: covering trachoma attached to the stem.**

Microscopic structures of *L. salicaria* aerial parts provide a useful and reliable criterion to the examination of powdered materials of the herb. The fragments of corolla were abundant in the powder of the plant aerial parts (Figure 1). The epidermal cells of corolla possessed thin, wavy walls with striated cuticle and also the calyx epidermis with anomocytic stomata and cicatrix were observed in the flower fragments. The fragments of the styles and stigmas were found in the powder, the structure of style was composed of thin-walled, longitudinally elongated cells. The epidermal cells of the apices of the stigmas were extended to form elongated, finger like papillae. Moreover, the occasional fragments of the fibrous layer of the anthers were composed of fairly large cells, while the abundant small spherical pollen grains were discovered with some furrows and a finely pitted exine. The cells of upper epidermis of the leaf were larger than lower epidermis; both were thin-walled in surface view (Figure 2). The walls of the epidermis cells were sinuous or wavy shapes and almost anomocytic stomata observed on both surfaces. The palisade cells underlying the upper epidermis were large and closely packed. The spongy mesophyll was composed of thin-walled spherical cells, in which the clusters of calcium oxalate were found. Covering trichomes were found abundantly on both epidermises, mainly along the veins on the lower epidermis. They were all conical and taper abruptly to a point at the apex, composed of one or two cells with slightly thickened and warty walls. The fragments of the stems consisted of large annularly, spiral, and reticulate thickened vessels accompanied with fibrous layer in some cases (Figure 3). The anomocytic stomata with cicatrix and covering trichomes were also observed in the stem parts of the plant same as other segments.

### Anti-diabetic activity

Anti-diabetic activity of the plant extract was tested using STZ-induced diabetic rats (Table 1). The results of this study revealed that the extract (15 g/kg) was significantly decreased blood glucose level. The plant extract (at dose of 15 g/kg) could reduce the blood glucose level by 12.6% and 7.3% during the second and third hours of administration, respectively. However, the rats treated with glibenclamide showed decreasing by 227% significantly after 2 h of administration. The plant extract with concentration of 12.5 g/kg was not effective to decrease the blood glucose level in the diabetic rats. The present results are in consistency with previous reports on hypoglycemic effect of *L. salicaria* extract suggesting augmentation of insulin secretion from langerhans cells [11,27]. Additionally, bioactive compounds of the plant have been previously isolated and identified as ursolic acid and oleanolic acid [6]. The mentioned triterpenes, purified from the *L. salicaria* extract, possess pentacylic structure with carboxylic functional group at C28, which forms an extensive hydrogen bond network. Previous results revealed that oleanolic acid administration to STZ-induced diabetic rats can significantly reduce the blood glucose level [28]. Moreover, these triterpenic acids are reported to induce a significant inhibition on protein tyrosine phosphatase 1B (PTP-1B), as an important enzyme, to influence insulin sensitivity through enhancing insulin receptor phosphorylation and stimulating glucose uptake [29,30]. Oleanolic acid was also able to enhance insulin secretion just the same as sulfonylurea at both basal and glucose-stimulated conditions in cultured cell [31]. Generally, *L. salicaria* extract is weakly able to decrease the blood glucose level probably by two mechanisms including high insulin secretion and sensitivity attributed to its active components.

**Table 1:** **Biological activity and phytochemical contents of *L. salicaria *extract**

**Various biological assays**	**Results (mean ± SE)**
Anti-diabetic^1^	−12.6%^2^
Anti-helicobacter	17 ± 0.08 mm^3^
Antioxidant	13.52^4^
Total flavonoids	5.8 ± 0.4 μg QE/mg EXT^5^
Total phenols	331 ± 3.7 μg GAE/mg EXT^6^
Total tannins	340 ± 2.3 μg TAE/mg EXT^7^
Total polysaccharides	21 ± 0.21 μg GE/mg EXT^8^

1: The plant extract was tested using STZ-induced diabetic rats, 2: % variation of glycemia in the second hour, 3: inhibition zone diameter (IZD), 4: free radical 50% inhibition (IC_50_), 5: quercetin equivalent in the extract (QE/EXT), 6: gallic acid equivalent in the extract (GAE/EXT), 7: tannic acid equivalent in the extract (TAE/EXT), 8: glucose equivalent in the extract (GE/EXT).

### Anti-*Helicobacter pylori* activity

*L. salicaria* traditionally has been used for some intestinal disorders. Therefore, in the present study its anti-*Helicobacter* activity was assessed against clinically isolated strain. In the preliminary evaluation, the plant extract exhibited low inhibitory activity against *H. pylori* isolated from patient with gastric disorder using disc diffusion method (Table 1). The extract of the plant (500 mg/mL) and Amoxicillin (1 μg/mL) inhibited the growth of the strain with zone diameter of inhibition as 17 ± 0.08 and 30 ± 0.04 mm, respectively. In addition, blood hemolysis was observed in the culture medium of the bacterium treated with the plant extract.

### Antioxidant activity

Value of IC_50_ for radical scavenging activity of the plant extract was also measured as13.5 μg/mL using DPPH method (Table 1). Antioxidant activities of vitamin E and BHA were tested as positive controls and IC_50_ values of them evaluated as 14.2, and 7.8 (μg/mL), respectively. The plant extract showed higher free radical inhibition than vitamin E. Previous preliminary studies have also exhibited antioxidant effect of the polar extracts of *L. salicaria* corroborating the results of this study [8,32].

### Phytochemical contents of the extract

The plant extract was subjected to chemical evaluation in the present study using different methods (Table 1). However, various genuine compounds (gallic acid, tannic acid and glucose) were employed to depict of standard curves in order to estimate the concentration of those components. Total contents of flavonoids, phenols, and tannins as well as polysaccharides were measured in the extract as 5.8 ± 0.4 μg QE/mg EXT, 331 ± 3.7 μg GAE/ mg EXT, 340 ± 2.3 μg TA/mg EXT, and 21 ± 0.2 μg GE/mg EXT, respectively. Various extracts of the plant were previously subjected to evaluation of flavonoids, phenols, and tannins contents together with antioxidant activity [32,33]. The results indicate that the phytochemical contents are found here in less concentration than those reported in previous study. This condition could be attributed to different plants origins and also application of diverse extraction methods.

## Conclusions

Taking together, microscopic structures of the plant powder, mentioned in this paper, could be an important identification method for determining the plant identity in the mixed herbal powders administered by traditional herbalist. The anomocytic stomata, conical trichomes with warty cell, and pollen grains were found as the main characteristics of the plant powder. Additionally, aqueous methanol extract (80%) of *L. salicaria* was found to exhibit low hypoglycemic effect in STZ-induced diabetic rats as well as anti-*Helicobacter pylori*. It seems that the triterpenic constituents, including oleanolic acid and ursolic acid, are responsible to induce sensitivity to the insulin [6,31]. Additionally, the results of preliminary studies revealed that the plant extract increased insulin secretion in the experimental animals [11,27]. Therefore, it could be concluded that the plant extract is able to decrease blood glucose level by two possible mechanisms: increasing insulin secretion and also augmenting of insulin sensitivity. In spite of the fact that, the lowering effect of blood glucose level due to the ingestion of plant extract is very light in comparison to the positive control, it might be important if the plant materials administrated with other anti-diabetic medicines. Although, the result of blood lowering effect of the plant extract is in consistency with previous studies, the mentioned effect of the plant is weaker than it consider as a blood glucose lowering agent. Moreover, the plant extract displayed excellent radical scavenging activity similar to vitamin E as a standard antioxidant, due to the high contents of phenolic compounds including tannins and flavonoids. The traditional use of this plant as an anti-inflammation factor could be attributed to the high content of its antioxidant chemicals.

## Competing interests

The authors declare that they have no competing interests.

## Authors’ contributions

AM: extraction, manuscript drafting and interpreting of data, MK: proposing of the plant, SS: participating in manuscript drafting and interpreting of data, EA: designing antidiabetic test and interpreting its data, MRM: performing plant extraction and anti-diabetic test, FV: phytochemical contents test, MM: participating in microscopic characterization, FS: *Helicobacter* test, AH: proposing of the plant. All authors read and approved the final manuscript.
