# Erratum: Biological activity and microscopic characterization of *Lythrum salicaria* L

**DOI:** 10.1186/s40199-014-0061-x

**Published:** 2014-10-09

**Authors:** Azadeh Manayi, Mahnaz Khanavi, Soodabeh Saeidnia, Ebrahim Azizi, Mohammad Reza Mahmoodpour, Fatemeh Vafi, Maryam Malmir, Farideh Siavashi, Abbas Hadjiakhoondi

**Affiliations:** Department of Pharmacognosy, Faculty of Pharmacy, Tehran University of Medical Sciences, Tehran, Iran; Traditional Iranian Medicine and Pharmacy Research Center, Tehran University of Medical Sciences, Tehran, Iran; Medicinal Plants Research Center, Faculty of Pharmacy, Tehran University of Medical Sciences, Tehran, Iran; Department of Pharmacology and Toxicology, Faculty of Pharmacy, Tehran University of Medical Sciences, Tehran, Iran; Med.UL, Faculty of Pharmacy, University of Lisbon, Lisbon, Portugal; Microbiology Department, Faculty of Sciences, University of Tehran, Tehran, Iran

## Text

After publication of this work [1] authors’ noted that the name of 3rd author (Soodabeh Saeidnia) was misspelled. The correct spelling is Soodabeh Saeidnia.
